# Supplementary material for: Relative Deprivation, Poverty, and Mortality in Japanese Older Adults: A Six-Year Follow-Up of the JAGES Cohort Survey
Source: Int J Environ Res Public Health. 2019 Jan 10;16(2):182. doi: 10.3390/ijerph16020182 (PMC6352140; doi:10.3390/ijerph16020182)
Supplement: Supplementary file 1 [file ijerph-16-00182-s001.pdf]

Supplementary Table 1

Original thirteen relative deprivation index; its prevalence and association with mortality.

Item	Category	%	Mortality Crude HR	(95%CI)
No television	No	98.1	ref.	
	Yes (+)	1.9	1.83*	(1.12 - 2.96)
No refrigerator	No	98.9	ref.	
	Yes (+)	1.1	2.01*	(1.11 - 3.65)
No air conditioner	No	95.5	ref.	
	Yes (+)	4.5	1.51*	(1.07 - 2.13)
No microwave oven	No	97.4	ref.	
	Yes (+)	2.6	1.43	(0.89 - 2.29)
No water heater	No	96.7	ref.	
	Yes (+)	3.3	1.42	(0.94 - 2.15)
No private bathroom	No	93.2	ref.	
	Yes (+)	6.8	1.45*	(1.08 - 1.96)
No private WC	No	95.9	ref.	
	Yes (+)	5.1	1.13	(0.77 - 1.65)
No private kitchen	No	93.5	ref.	
	Yes (+)	6.5	1.30	(0.95 - 1.79)
Dining room separated from	Yes	86.6	ref.	
bedroom	No (+)	13.4	1.14	(0.90 - 1.79)
No ceremonial dress	No	98.6	ref.	
	Yes (+)	1.4	1.84*	(1.04 - 3.27)
No telephone	No	96.8	ref.	
	Yes (+)	3.2	1.13	(0.70 - 1.80)
Absence from family ceremo-	No	94.6	ref.	
nial occasions	Yes (+)	5.4	1.65**	(1.21 - 2.26)
Cut-off of essential services in	No	98.9	ref.	
the past year	Yes (+)	1.1	2.12*	(1.17 - 3.85)

** p<.01 * p<.05 HR: Hazard ratio, 95%CI: 95% confidential interval.
(+) is related to relative deprivation.
